# MCGNet^+^: an improved motor imagery classification based on cosine similarity

**DOI:** 10.1186/s40708-021-00151-3

**Published:** 2022-02-01

**Authors:** Yan Li, Ning Zhong, David Taniar, Haolan Zhang

**Affiliations:** 1 Zhejiang University, Hangzhou, China; 2 Maebashi Institute of Technology, Maebashi, Japan; 3 Monash University, Melbourne, Australia; 4 Ningbo Institute of Technology, Zhejiang University, Ningbo, China

**Keywords:** Graph convolutional networks, Electroencephalography (EEG), Brain–computer interfaces (BCI)

## Abstract

It has been a challenge for solving the motor imagery classification problem in the brain informatics area. Accuracy and efficiency are the major obstacles for motor imagery analysis in the past decades since the computational capability and algorithmic availability cannot satisfy complex brain signal analysis. In recent years, the rapid development of machine learning (ML) methods has empowered people to tackle the motor imagery classification problem with more efficient methods. Among various ML methods, the Graph neural networks (GNNs) method has shown its efficiency and accuracy in dealing with inter-related complex networks. The use of GNN provides new possibilities for feature extraction from brain structure connection. In this paper, we proposed a new model called MCGNet^+^, which improves the performance of our previous model MutualGraphNet. In this latest model, the mutual information of the input columns forms the initial adjacency matrix for the cosine similarity calculation between columns to generate a new adjacency matrix in each iteration. The dynamic adjacency matrix combined with the spatial temporal graph convolution network (ST-GCN) has better performance than the unchanged matrix model. The experimental results indicate that MCGNet^+^ is robust enough to learn the interpretable features and outperforms the current state-of-the-art methods.

## Introduction

Brain–computer interface (BCI) technology has drawn much attention globally due to its significant meaning and extensive applications [1]. It enables their users to interact with the machine through the brain signals [2], such as the task of converting the psychological imagination of motion into a command [3], which can be utilized to help people with disabilities as a rehabilitation device [4] and could be considered the only way for people with motor disabilities to communicate [5]. The motor imagery classification based on the features extracted from the EEG imagination data of moving the body parts without actual movement, but the feature extraction process often relies heavily on prior knowledge to exclude certain features [6]. Consequently, more robust feature extraction techniques will continue to drive the development of BCI technologies.

A typical brain–computer interface system consists of four main processes [7]: brain-electric raw data acquisition, data preprocessing, feature extraction and feature classification. The previous studies show that the feature extraction and classification are two important phases, which determine whether the system is effective or not. The feature extraction process is designed to describe EEG signals by relevant values [8], and features should contain the information embedded in the original EEG signals while filtering out the noise and other irrelevant information. The classification phase is critical because an efficient classifier can take advantage of as many extracted features as possible and greatly improve the accuracy of the classification. The motor imagery classification is an EEG-based task that focuses primarily on the feature extraction and classification, which have been studied extensively in previous work. Some research highlights two most common types of features that include frequency band power features and time point features [9], both of which benefit from extracting zone after spatial filtering [10]. Principal component analysis (PCA) and independent component analysis (ICA) are two classic unsupervised spatial filter methods [11], supervised spatial filters include the common spatial patterns (CSP) and filter bank common spatial patterns(FBCSP) [12]. In terms of the classifiers for motor imagery task, many state-of-the-art methods have been proven effective, such as linear discrimination analysis (LDA) and support vector machine (SVM) [13].

Nowadays, the deep learning methods have been efficiently applied to various areas. Much recent work has explored the application of deep learning to EEG-based analytical tasks [14]. The deep learning methods improve the analytical efficiency and accuracy and provide end-to-end learning for EEG-based tasks, such as sleep stage detection, anomaly detection, motor imagery classification and so on [15]. In spite of the typical deep learning methods, such as convolution networks, can learn from the raw data without manual feature extraction, they still have some major limitations. For instance, typical deep learning methods require large datasets to train the models, which can be a disadvantage for EEG-based tasks because the collection of EEG data usually costs a lot. In addition, EEG datasets represent the unique characteristics of an individual, and the data collected from different areas of the brain. Therefore, the spatial connection between the EEG data cannot be ignored. However, existing methods including recent deep learning methods are unable to effectively learn the connections between different brain regions [16].

Graphs are the most appropriate data structure for brain connections; and graph neural networks (GNNs) has been shown to be effective in classifying graph structures [17]. The core idea of GNNs is to update each node’s embedding iteratively through aggregating the representations of its neighbors and itself. The EEG channels could be represented as nodes in the graph and the connections between the channels correspond to the edges of the graph, but the graph convolutional networks need adjacency matrix to be given in advance which is the representation of the graph connection [18], so determining a suitable brain map structure is still a challenge due to the limitations of cognition of brain structure. And there are some methods that could be used to generate the adjacency matrix, we could utilize the position to calculate the distance between the electrodes as the degree of correlation or utilize the features collected from the electrodes to calculate the correlations. Moreover, the collection of EEG data is usually in chronological order, so in addition to spatial characteristics the temporal characteristics also need to be taken into account.

In this paper, we proposed a novel model called MCGNet$^{+}$ based on the our proposed MutualGraphNet, combined the spatial–temporal filter and graph convolutional networks to learn the temporal and spatial characteristics, which achieved robust performance on the motor imagery classification tasks. The contributions of this paper are as follows:

- The model could realize end-to-end learning. Furthermore, the model is specially designed to adapt to the characteristics of EEG data, so it could be able to utilize the features to a great extent.
- For the first time, we use mutual information to generate the initial adjacency matrix and use cosine similarity to update the adjacency matrix dynamically, and achieve better performance.
- Experimental results demonstrate that the newly proposed model has better performance than state-of-the-art methods.

## Related work

A motor imagery classification task is of great significance for people with disabilities. Numerous works have been done to improve classification performance. In earlier studies, traditional machine learning methods were commonly used for motor imagery classification task, such as support vector machine (SVM), K-Nearest-Neighbor (KNN) and artificial neural network (ANN) are frequently used [19], but these traditional methods have limited performance on EEG-based classification tasks. Currently, the deep learning methods are utilized in EEG-based classification tasks, Deep Belief Network (DBN) [20] was proposed to manually extract features from the channels then feed them into the network. Convolutional Neural Networks(CNN) could automatically learn features from EEG data and perform better than DBN due to their regular structure and the degree of ambiguity of the translational structure [21]. Two CNN models were specially designed for motor imagery classification called Shallow ConvNets and Deep ConvNets [14], both of them have better performance than the state-of-the-art methods. Then another CNN model called EEGNet [15] was proposed, which utilizes the Depthwise and Separable convolutions to replace the traditional convolutions for the motor imagery task that have better performance than the ConvNets.

The CNN models can effectively extract the local patterns of data, but it can only be applied to the standard grid data [22], graph convolutional networks have been proven to have better performance on the graph structure data. Much has been done to improve the performance of the graph convolutional networks. So far, GCNs have been applied in many fields, the spatial–temporal graph convolution network (ST-GCN) [23] is proposed to learn the dynamic graphs for the human action recognition tasks, the spatiotemporal multi-graph convolution network (ST-MGCN) [24] is proposed for ride-hailing demand forecast which encodes the non-Euclidean correlations among regions into multiple graphs, GraphSleepNet [16] based on spatial–temporal convolution network (ST-GCN) is proposed for automatic sleep stage classification. When using GCNs, the connection relationship between each electrode need to be given as a prior knowledge, in other words, the adjacency needs to be calculated as input.

There are different methods that can be used to generate the adjacency matrix, the distance between two electrodes can be used directly to represent the degree of correlation between electrodes and there are many different vector distance calculation methods, such as the euclidean distance [25] which only need the physical position of the electrodes, the Chebyshev distance [26] is defined as the maximum difference between two vectors in any coordinate dimension, hamming distance, Manhattan distance and so on. Furthermore, we can use the correlations of vectors to determine the degree of relevance of the different channels, such as cosine similarity [27] that calculates the similarity relationship between the characteristics of different electrode channels, Pearson correlation that evaluates the linear relationship between two continuous variables, Spearman correlation that evaluates the monotonic relationship between two continuous variables, Kendall correlation, Point-Biserial correlation and so on. Also, we could use some machine learning methods, such as the information gain [28] that evaluates the gain of each variable in the context of the target variable and mutual information is the name given to information gain when applied to variable selection that calculates the statistical dependence between two variables.

Motivated by the studies mentioned above, considering the graph structure and the dynamic spatial–temporal characteristic of the EEG data as well as the graph structure of different motor imagery could be different, the traditional GCNs models may not be optimal for EEG-based motor imagery classification task. Thus, we propose the novel model to best suit the characteristics of EEG data which uses the mutual information to generate the initial adjacency matrix and use the cosine similarity to update the adjacency matrix after each iteration.

## Preliminaries

**Fig. 1:**
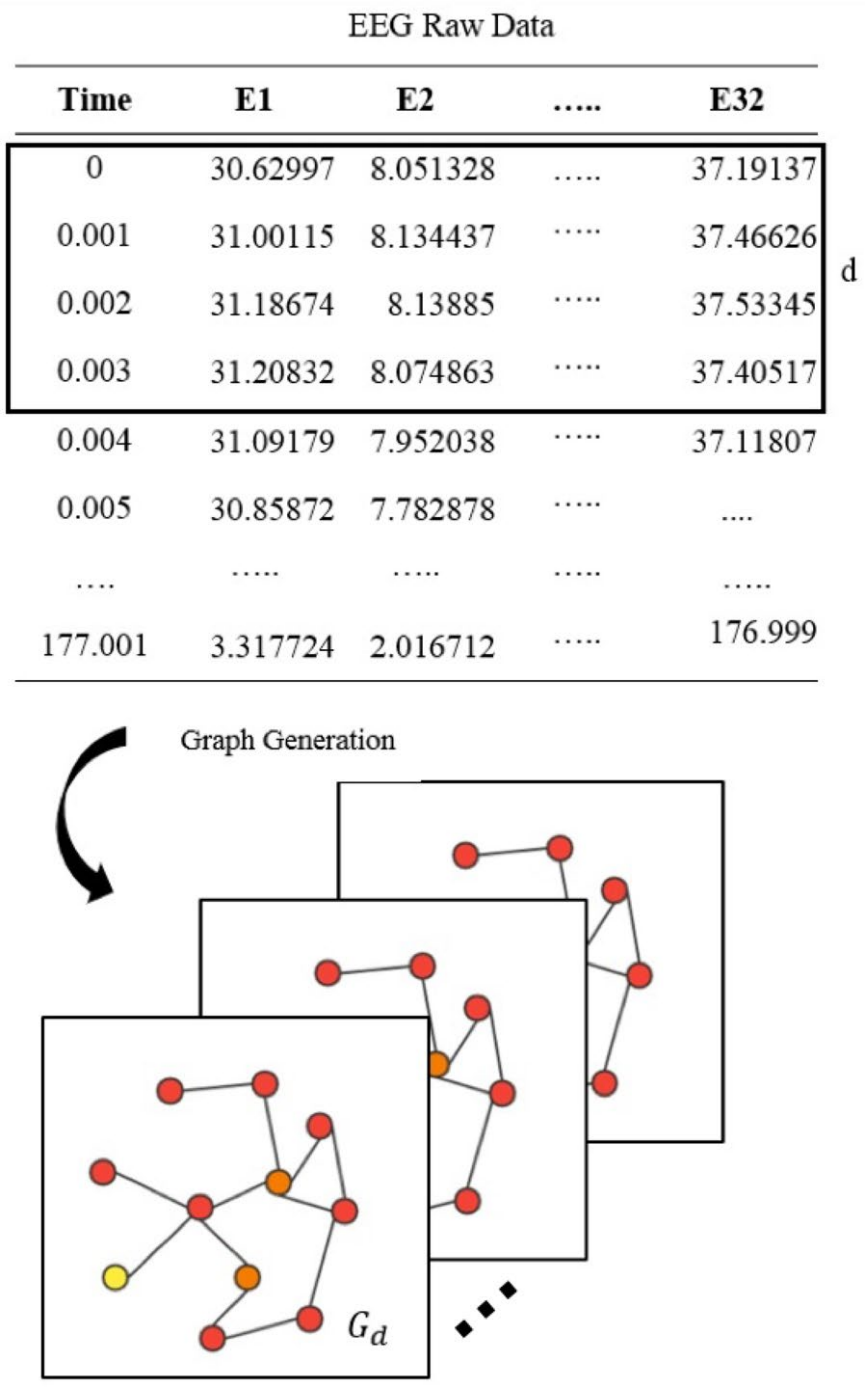
The structure generation of EEG data, where the data at the range of time *d* forms a graph

In this study, the EEG data could be defined as an undirected graph $G = (V, E, A)$ , where *V* is a finite set of $|V| = N$ nodes and *N* represents the number of the EEG data channel; *E* is a set of edges, indicating the connectivity between different channels; *A* represents the adjacency matrix of graph *G*. Figure 1 shows how the graph is generated from the EEG raw data.

The recorded EEG signals are divided into several labeled segments called trials, the *d*th trial can be denoted as $X^d = (x_t^1, x_t^2, ..., x_t^N)^F \in {\mathbb {R}}^{N \times F}$, where *N* denotes the number of the EEG electrodes and *F* denotes the values of all nodes within the time steps *t*. The dataset can be described as $D = {(X^{1}, y^{1}) , (X^{2},y^{2}) , ..., (X^{L},y^{L})}$, *L* denotes the number of the trials and *y* represents the label corresponding to the trial, there are four motor imagery categories including left hand, right hand, feet and tongue, so the label can be denoted by 0–3, respectively. The goal of the task is to learn the mapping relationship between the EEG data and the motor imagery categories represented as labels and the problem can be defined as: given a input trial $X^i\in {{\mathbb {R}}^{N \times F}}$, $0<i<L$ identify the corresponding label $y^i$.

## Methodology

**Fig. 2:**
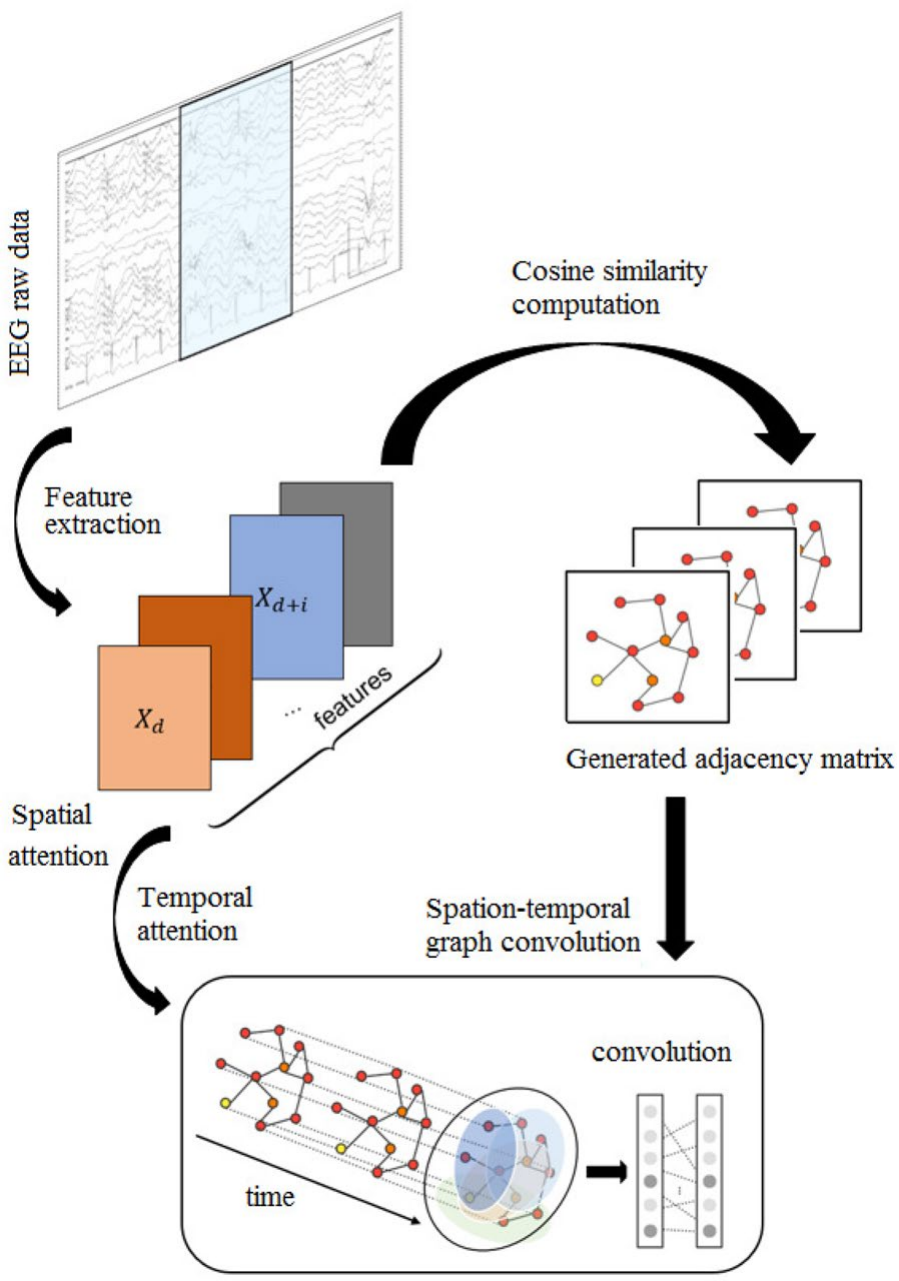
The overall structure of the proposed model consists of three parts: the feature extraction and the mutual information computation part, the spatial–temporal attention mechanism part and spatial–temporal graph convolution part

The overall framework of the model proposed in this paper is presented in Fig. 2, it includes three main parts: feature extraction and adjacency matrix generation part, spatial–temporal attention part and spatial–temporal graph convolution part. Spatial–temporal attention part puts more attention on the more valuable spatial–temporal information, then spatial–temporal graph convolution part extracts both spatial and temporal features. And the complete algorithm can be seen as follows:

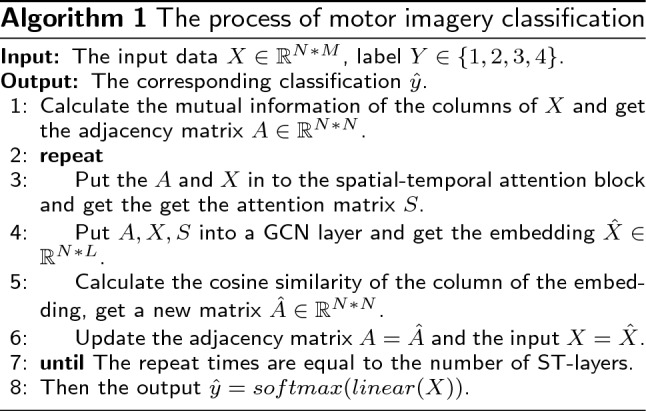



### Adjacency matrix generation

#### Relevance calculate methods

The relevance of different electrodes can be obtained through calculating the correlations or the information gain of the features of the electrodes, and in this paper we calculate the relevance of different electrodes over all the electrodes. The correlations of different channels can be represented by the distances of the channels. The euclidean distance of the electrodes can be represented as:

1 $$\begin{aligned} \rho = ((x_2 - x_1)^2 +(y_2 -y_1)^2 +(z_2 -z_1)^2)^{1/2}. \end{aligned}$$

The euclidean distance can be understood as the straight-line distance between two points, but the electrodes are distributed on the surface of the cerebral cortex, so it is not suitable to directly express the relationships between the electrodes. The Chebyshev distance is defined as the maximum difference between two vectors in any coordinate dimension, it is the maximum distance along an axis, and the Chebyshev distance of the electrodes can be denoted as:

2 $$\begin{aligned} \rho = \mathrm{{max}}(|x_2 -x_1|,|y_2 - y_1|, |z_2 - z_1|). \end{aligned}$$

The calculation of the distances of the electrodes only utilizes the positions of the electrodes, we can also use the features of the electrodes to obtain the correlations. The cosine similarity of two vector can be defined as:

3 $$\begin{aligned} cos(x,y) = \frac{x.y}{\Vert x\Vert \Vert y\Vert }. \end{aligned}$$

However, the cosine similarity does not consider the magnitude of the vectors, but only consider the directions. The Jacquard index, also known as the intersection ratio and Jacquard similarity coefficient, can be used to compare the similarity and diversity of sample sets:

4 $$\begin{aligned} J(x_1,x_2)=\frac{|x_1\bigcap x_2|}{|x_1|+|x_2|-|x_1\bigcap x_2|}. \end{aligned}$$

One of the main disadvantage of the Jacquard index is that it is greatly affected by the size of the data. Large datasets have a great impact on the index, because it can significantly increase the union while maintaining similar intersection. Moreover, we could use information gain between the feature vectors to obtain the degree of relevance, information gain is calculated by comparing the entropy of the dataset before and after a transformation. The mutual information calculates the statistical dependence between two variables and is the name given to information gain when applied to variables selection.

#### Adjacency matrix update

In order to make full use of and adjust the input prior knowledge in time according to the embedding learned by GCNs, we use the mutual information to generate the initial adjacency matrix and use the cosine similarity to update the adjacency matrix during the training process.

Mutual information (MI) [29] is used to indicate whether there is a relationship between two variables and the strength of the relationship. The mutual information of two variables *X* and *Y* can be defined as:

5 $$\begin{aligned} I(X,Y) = \sum _{x\in X}\sum _{y\in Y}p(x,y){\rm{log}}\frac{p(x,y)}{p(x)p(y)}. \end{aligned}$$

Mutual information is related to entropy, which is the expected or mean value of the information of all variables. The entropy of *X* is defined as:

6 $$\begin{aligned} \begin{aligned} \quad H(X)&= \sum _{x\in X}P(x){\rm{log}}\frac{1}{P(x)} \\&= - \sum _{x\in X}P(x){\rm{log}}P(x) = -E{\rm{log}}P(X). \\ \end{aligned} \end{aligned}$$

Then MI of *X* and *Y* can be computed by the equations:

7 $$\begin{aligned} \begin{aligned} \quad I(X,Y)&= H(X) + H(Y) -H(X,Y) \\&= H(X)-H(X|Y) = H(Y)-H(Y|X) \\ H(X,Y)&= \sum _{x \in X}\sum _{y \in Y}p(x,y){\rm{log}}\frac{1}{p(x,y)} = -E{\rm{log}}P(X,Y) \\ \quad H(Y|X)&= \sum _{x \in X}\sum _{y \in Y}p(x)p(y|x){\rm{log}}\frac{1}{p(y|x)} \\&= -E{\rm{log}}P(Y|X), \end{aligned} \end{aligned}$$

where *H*(*X*, *Y*) is the joint entropy of *X* and *Y*, and *H*(*Y*|*X*) is the conditional entropy that *X* is given in advanced. Thus, *I*(*X*, *Y*) is the reduction in the uncertainty of the variable *X* by the knowledge of another variable *Y*, equivalently, it represents the amount of information that *Y* contains about *X*.

Considering the features of EEG data $X = \{x^1, x^2,...,$ $x^N\}\in {\mathbb {R}}^{N\times F}$, we could compute the mutual information $m_ij$ of $x^i$, $x^j$ and use it as the weight of the connection of $x^i,x^j$, then we could generate a $N\times N$ weight matrix which could be used as the input adjacency matrix of the graph convolution networks. In our proposed work [30], we kept the initial adjacency matrix unchanged during the training process. However, on embedding changes after each iteration, we update the adjacency matrix after each iteration synchronously to improve the performance of the model. Here, we compute the cosine similarity of two columns of the embedding as the weight of the adjacency matrix. The cosine distance of two vector *x*, *y* is defined as:

8 $$\begin{aligned} cos(x,y) = \frac{x.y}{\Vert x\Vert \Vert y\Vert }. \end{aligned}$$

**Fig. 3:**
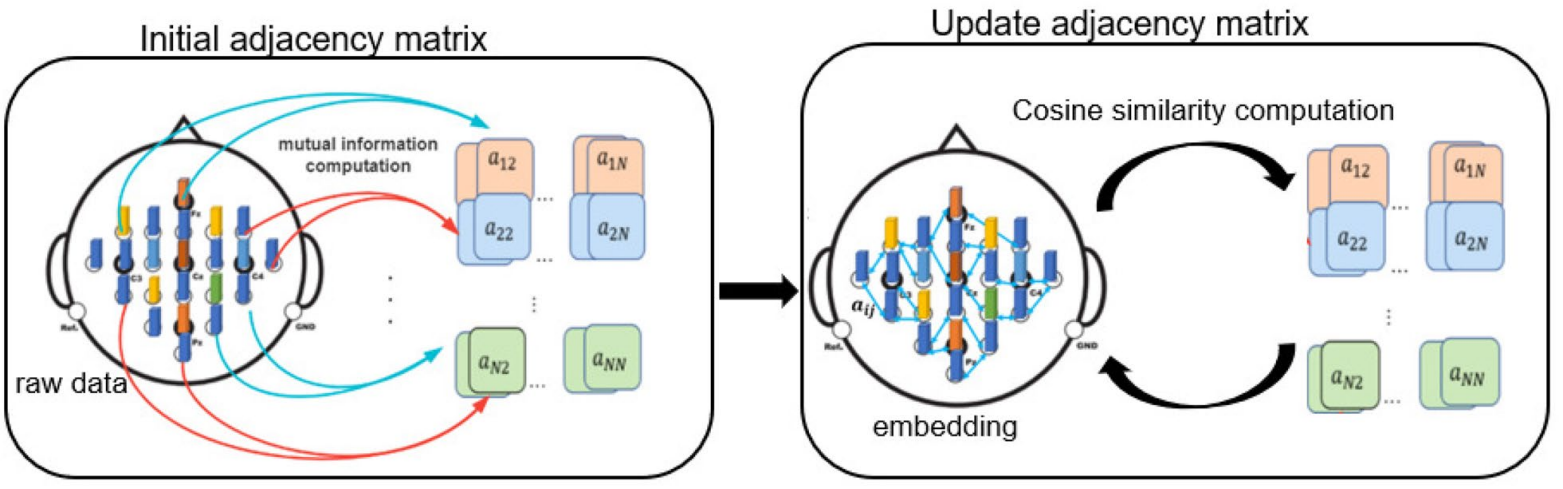
The process of generating and updating the adjacency matrix

The updated weight can be defined as:

9 $$\begin{aligned} a_{i,j}^{l+1} = \frac{e_{i}^{l}.e_{j}^{l}}{\Vert e_{i}^{l}\Vert \Vert e_{j}^{l}\Vert }, \end{aligned}$$

where the $a_{i,j}^{l+1}$ denotes the element of the *i*th row and *j*th column of the adjacency matrix at the $l+1th$ iteration, and $e_{i}^{l}, e_{j}^{l}$ represents the *i*th, *j*th column of the embedding at *l*th iteration. The process of generating and updating the adjacency matrix can be seen in Fig. 3.

### Spatial–temporal attention

The spatial–temporal attention mechanism could capture the dynamic spatial and temporal correlations of the motor imagery network. In the spatial dimension, the activities of one brain region has influence on other brain regions and generally different brain activities convey different information, so the dynamic spatial–temporal capture mechanism is required. We use a spatial attention mechanism [31], which could be represented as:

10 $$\begin{aligned}S &= V_p*\sigma ((\chi ^{(r-1)}W_1)W_2(W_3\chi ^{(r-1)})^T +b_{p}), \\ \quad S^{'}_{i,j} &= \frac{\mathrm{{exp}}(S_{i,j})}{\sum \limits ^N_{j=1}\mathrm{{exp}}(S_{i,j})}, \end{aligned}$$

where *S* denotes the spatial attention matrix, which is computed by current layer. $V_p, b_p \in {\mathbb {R}}^{N\times N}$, $\chi ^(r-1) = (X_1,X_2, \cdots , X_{T_{r-1}} \in {\mathbb {R}}^{N\times C_{r-1}\times T_{r-1}}$ $C_{r-1}$ is the number of channels of the input data in the $r^th$ layer. $W_1 \in {\mathbb {R}}^{T_{r-1}}, W_2 \in {\mathbb {R}}^{C_{r-1}\times T_{r-1}}, W_3 \in {\mathbb {R}}^{C_{r-1}}$, $S_{i,j}$ in *S* represents the correlation strength between node *i* and *j*, then a softmax function is used to normalize the attention weights. Combining the adjacency matrix and the spatial attention matrix, the model could adjust the impacting weights between nodes dynamically.

In the temporal dimension, there are correlations during each motor imagery trial, such that the brain waves are transmitted in the cerebral cortex and the active areas of the brain will change over time, so the collected EEG data also changes over time. Therefore, a temporal attention is utilized to capture dynamic temporal information. The temporal attention mechanism is defined as:

11 $$\begin{aligned}E &= V_e*\sigma (((\chi ^{(l-1)})^{T}M_1)M_2(M_3\chi ^{(l-1)})+b_q), \\\quad E^{'}_{m,n} &= \frac{\mathrm{{exp}}(E_{i,j})}{\sum \limits ^{T_{r-1}}_{j=1}\mathrm{{exp}}(E_{i,j})}, \\ \end{aligned}$$

where $V_e, b_q \in {\mathbb {R}}^{T_{l-1}\times T_{l-1}}$, $M_1 \in {\mathbb {R}}^N, M_2 \in {\mathbb {R}}^{C_{l-1}\times N}$, $M_3 \in {\mathbb {R}}^{C_{l-1}}$, $E_{m,n}$ denotes the strength of the correlation between motor imagery network *m*, *n*, and *E* is normalized by the softmax function, so the temporal attention matrix can be directly applied to the input.

### Spatial–temporal graph convolution

The spatial–temporal convolution consists of a graph convolution in the spatial dimension and a normal convolution in the temporal dimension, which could extract both the spatial features and the temporal features.

The spatial features are extracted by aggregating information from neighbor nodes; we use graph convolution to extract the spatial features. The graph convolution is based on Laplacian matrix and Fourier transform, the graph Laplacian can be defined as:

12 $$\begin{aligned} L = I - D^{-1/2}AD^{-1/2}, \end{aligned}$$

where $A\in {\mathbb {R}}^{N\times N}$ is the adjacency matrix associated with the graph, $D \in {\mathbb {R}}^{N\times N}$ is the diagonal degree matrix, $I\in {\mathbb {R}}^{N\times N}$ is the identity matrix. *L* is a real symmetric positive semidefinite matrix, it can be decomposed as $L = U\Lambda U^T$ and $\Lambda \in {\mathbb {R}}^{N\times N}$ is the diagonal matrix of eigenvalues that represent the frequencies of their associated eigenvectors. Let $x \in {\mathbb {R}}^n$ be a signal defined on the vertices of a graph *G*, the graph Fourier transform of the signal is defined as x̂ = $U^Tx$. The graph convolution uses the linear operators that diagonalize in the Fourier domain to replace the classical convolution operator, the graph convolution can be defined as:

13 $$\begin{aligned} g_\theta (L)x = g_\theta (U\Lambda U^T)x = Ug_\theta (\Lambda )U^Tx, \end{aligned}$$

where $\theta$ is a vector of Fourier coefficients, $g_\theta$ is the filter that could reduce the computational complexity, $g_\theta$ can be approximated by a truncated expansion in the terms of Chebyshev polynomials [32]:

14 $$\begin{aligned} g_{\theta }({\Lambda }) = \sum ^{k-1}_{p=0}{\theta }_{p}T_{p}({\tilde{\Lambda }}), \end{aligned}$$

where *k* is the order of the Chebyshev polynomials, $\theta _p \in {\mathbb {R}}^k$ is the vector of Chebyshev coefficients, $T_{p}({\tilde{\Lambda }})\in {\mathbb {R}}^{N\times N}$ is the Chebyshev polynomial of order *k* and ${\tilde{\Lambda }} = 2{\Lambda }/{\lambda }_{max} -I$ ranges in $[-1,1]$. Then the *jth* output feature can be calculated as:

15 $$\begin{aligned} y_i = \sum ^{F_{in}}_{i=1}g\theta _{i,j}(L)x_i, \end{aligned}$$

where $x_i$ denotes the *i*th row of input matrix, $F_{in}$ equals to the input dimension, the outputs are collected into a feature matrix $Y = [y_1,y_2,\ldots ,y_{F_{out}}] \in {\mathbb {R}}^{N \times F_{out}}$. In this work, we generalize the above definition to the nodes with multiple channels, the *l*th layer’s input is $X^{(l-1)} = (x_1,x_2,\ldots ,x_{(T_{l-1})}) \in {\mathbb {R}}^{N\times C_{l-1}\times T_{l-1}}$, $C_{(l-1)}$ denotes the channel’s number and $T_{l-1}$ denotes the *l*th layer’s temporal dimension.

After the graph convolution having captured the neighboring information for each node in the spatial dimension, a standard convolution layer is used in the temporal dimension, we use a standard two-dimension convolution layer to extract the temporal information, the *r*th convolution layer could be defined as:

16 $$\begin{aligned} \chi ^{(r)}_h = {\rm ReLU}(\Phi *({\rm ReLU}(g_\theta * G {\hat{\chi }}^{(r-1)}_{h}))), \end{aligned}$$

where $\Phi$ is the parameter of the temporal dimension convolution kernel, and $*$ represents the convolution operation, ReLU is the activation function.

## Experiment

In order to evaluate the effectiveness of our model, we carried out the comparative experiments on a public dataset BCI Competition IV dataset 2a(SMR) for motor imagery task.

### Dataset description

**Fig. 4:**
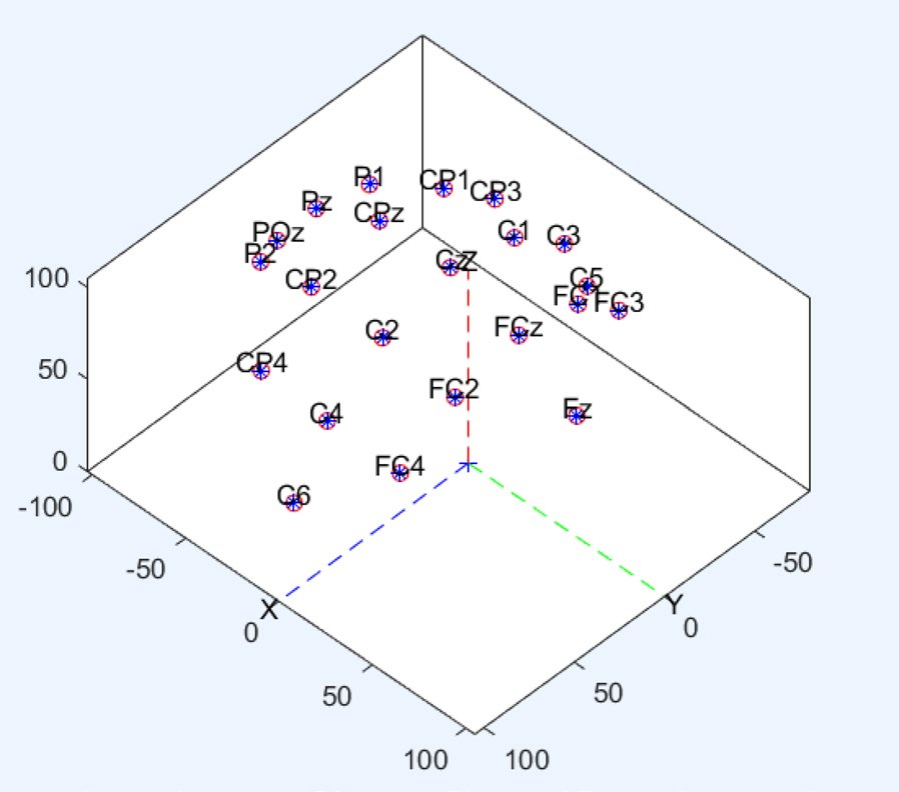
The distribution of the electrodes in 3D space

The BCI Competition IV dataset 2a consists of EEG data from nine subjects, there are two sessions recorded, one for training and the other one for testing. Each session includes 288 trials, which are recorded with 22 EEG electrodes and 3 electrooculogram channels, we only utilize 22 EEG channels in this experiment and the distribution of the EEG electrodes can be seen in Fig. 4. There are four types of labels in this dataset, corresponding to movements of the left hand, right hand, feet and tongue.

The original dataset is sampled at 250 Hz and bandpass-filtered between 0.5 Hz and 100 Hz, and we low-pass filter the dataset to 4–40 Hz. Also in our experiment, we set the length of each trial to 4.5 s which starts from 500 ms before the start cue of each trial until to the end cue, then we extract 11 differential entropy features (DE) for each channel and double fold the features to make it have the same shape as the adjacency matrix, and combine the two as the input of the graph convolutional network, then we standard scale the data to make it suitable for the machine learning model. To show the effectiveness of our proposed model learning from the raw data and ensure the model could be used for wider range of tasks, the raw EEG data have not undergone more preprocessing.

### Experiment settings

We compare our model with some state-of-the-art methods as well as the proposed MutualGraphNet, the baseline methods are listed as follows:

1. Filter Bank Common Spatial Patterns (FBCSP) [12]: it extracted the band power features of EEG, then use the features to train the classifier to predict the labels.
2. Shallow ConvNet [14]: an end-to-end learn method, which uses convolutional networks to do all the computations.
3. Deep ConvNet [14]: it has more convolution-pooling blocks and is much deeper than Shallow ConvNet.
4. EEGNet [15]: it uses the depthwise and separable convolution and has two convolution-pooling blocks.

In addition to the above baseline methods, we also conducted a comparison between the proposed method in this paper and the traditional machine learning methods including support vector machine (SVM) [33] and random forest (RF) [34].

In order to prove that the model can effectively extract features and has the ability to eliminate the influence of individual differences, we no longer conduct experiments on each subject separately, we mixed the experimental data of nine subjects, and a total 2592 training trials and 2592 testing trials, and we use fourfold cross-validation to evaluate the performance. Since the training set is not big enough, in order to reduce the impact of over-fitting, we adopt a loss flooding strategy [35] during the training process, which is defined as: ${\tilde{R}}(g) = |{\tilde{R}}(g) - b| + b$ and ${\tilde{R}}(g)$ is the loss of the model, *b* is a constant called loss flooding level, here we set *b* as 0.5. All these experiments are performed on a single Nvidia RTX3090 32GB GPU and the hyper-parameters are shown in Table 1.

**Table 1:** The hyper-parameters of the model and their corresponding values

Hyperparameter	Value
Learning rate	9.6e–4
Learning rate decay	0
Dropout rate	0.5
Optimizer	Adam
L1, L2 regularization	0.002, 0.001
Training epochs	500
Batchsize	32
Chebyshev polynomial	2

As for the baseline methods, in order to evaluate the performance of the models more reasonably, we use 250 Hz sampling 4.5 s EEG data for all experiments. Since that the EEGNet [15] used the 128 Hz resampled data to conduct experiment in the original paper, so we double the lengths of temporal kernels and average pooling size of the original model for double sampling rate to better adapt the input which proven to have better performance than the original model. In response to changes in the length of the sampling time, we also adjusted the parameters of each model accordingly, conducted experiments and selected the best model performance. The training parameters of other baseline methods are the same as in the paper [15].

### Results and discussion

We compare our model with the six baseline methods on SMR, we use the accuracy, F1-score and precision as the evaluation metrics to evaluate the performance of the models. Table 2 shows the performance of the different models on the SMR dataset, the results show that our model performs better compared to the other baseline methods and the proposed MutulaGraphNet.

**Table 2:** The performance comparison of the state-of-the-art approaches on the SMR dataset

Model	Accuracy	F1-score	Precision
SVM	0.3488	0.3485	0.3486
Deep ConvNet	0.3507	0.3191	0.4148
FBCSP	0.3511	0.3366	0.3714
RF	0.4008	0.3996	0.4004
EEGNet	0.4616	0.4838	0.5095
Shallow ConvNet	0.4857	0.4789	0.4978
MutualGraphNet (ours)	0.5190	0.5175	0.5208
MCGNet$^{+}$ (ours)	**0.5227**	**0.5239**	**0.5278**

For the traditional methods, the random tree (RF) has better performance than the support vector machine (SVM), but both of them are not good enough. The FBCSP cannot extract and utilize complex features in multi-subject tasks [36], though it has good performance in single-subject tasks. And the results show that the traditional machine learning methods cannot learn the complex features well, the deep learning models EEGNet and ShallowConvNet all outperform the traditional methods which demonstrate the effectiveness of deep convolutional neural networks for EEG-based classification tasks. However, the performance of DeepConvNet demonstrates that the deeper convolutional network does not work better. The values in bold shown in Table 2 indicate that our model (MCGNet) outperforms conventional methods in accuracy, F1-score and precision.

**Fig. 5:**
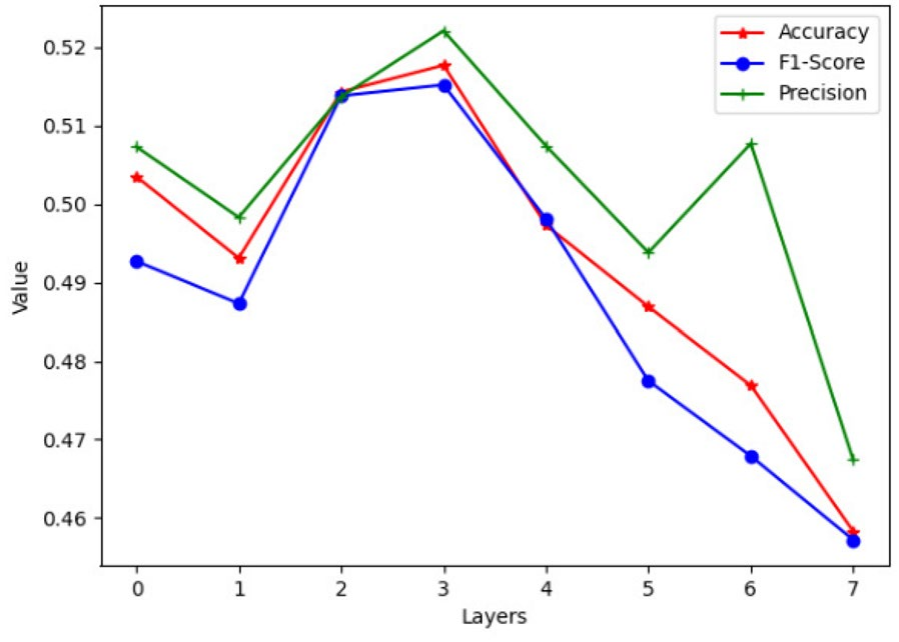
Performance of the proposed model with different ST-GCN layers

In order to evaluate the effect of the depth of network, we study the impact of the layers of ST-GCN in Fig. 5. The horizontal axis in Fig. 5 represents the layers of ST-GCN and the vertical axis represents the corresponding performance of the model. The results show that the MCGNet$^{+}$ with more ST-GCN layers does not work better; the best performance is achieved with 4 layers and with the increasing number of layers the performance gets worse. That is because the increase in the number of layers leads to an increase in training parameters, but the training dataset is too small to train the model with more parameters.

In this paper, we extract differential entropy (DE) feature as the input of the model, and in EEG-based tasks there are other five different features [37]: power spectral density (PSD), differential asymmetry (DASM), rational asymmetry (RASM), asymmetry (ASM) and differential caudality (DACU) features from EEG. The DASM and RASM can be expressed as:

17 $$\begin{aligned} {\mathrm{DASM}} = {\mathrm{DE}}(X_{\mathrm{{left}}}) - {\mathrm{DE}}(X_{\mathrm{{right}}}), \end{aligned}$$

18 $$\begin{aligned} {\mathrm{RASM}} = {\mathrm{DE}}(X_{\mathrm{left}})/ {\mathrm{DE}}(X_{\mathrm{right}}). \end{aligned}$$

ASM features are the direct concatenation of DASM and RASM features. DCAU features are the difference between DE features of frontal–posterior electrodes, which can be defined as:

19 $$\begin{aligned} {\mathrm{DCAU}} = {\mathrm{DE}}(X_{\mathrm{frontal}})-{\rm DE}(X_{\mathrm{posterior}}). \end{aligned}$$

We also evaluate the performance of our models on these features. All the experiments are performed with fourfold cross-validation and the training settings are the same as above.

**Table 3:** The performance of models for different features

Model	Feature	Accuracy	F1-score	Precision
MCGNet$^{+}$	PSD	0.2716	0.2695	0.2726
	DSAM	0.4124	0.4049	0.4052
	ASM	0.4039	0.3842	0.3877
	ASDM	0.3973	0.3881	0.3881
	DCAU	0.4375	0.4381	0.4435
	DE	**0.5227**	**0.5239**	**0.5278**
MutualGraphNet	PSD	0.2604	0.2286	0.2595
	DSAM	0.3646	0.3523	0.3541
	ASM	0.3815	0.3820	0.3879
	ASDM	0.3811	0.3777	0.3764
	DCAU	0.4162	0.4144	0.4191
	DE	**0.5190**	**0.5175**	**0.5208**

The results are presented in Table 3, the PSD feature still has the worst performance and the DE feature outperforms the other features. The DCAU feature also achieves comparable performance, but ASDM and DSAM feature contain less information which leads to limited performance. All the features have better performance with the new model, which indicated the effectiveness of the newly proposed method. Moreover, the results indicate that there exists some kind asymmetry of the brain which has discriminative information and our knowledge of the human brain is still very limited, the deeper understanding of brain is still required to obtain more effective and valuable information from EEG data. The values in bold shown in Table 3 indicate that DE feature in both our models (MCGNet and MutualGraphNet) outperforms other features in accuracy, F1-score and precision. The new approach is compared with the several different adjacency matrixes that we designed:

1. KNN: for each channel, select the nearest *N* channels to establish a connection.
2. The Euclidean distance(ED): according to the actual distance of each electrode on the brain, select adjacent points to establish a connection.
3. Random: randomly select channels and establish connections between channels.
4. Mut_Euclidean : use the Euclidean distance to establish connections and calculate the mutual information.
5. Mut_KNN: use KNN to establish connections and calculate mutual information between connected channels.
6. Mut_ED: use the Euclidean distance to confirm connection and calculate mutual information between connected channels.

**Fig. 6:**
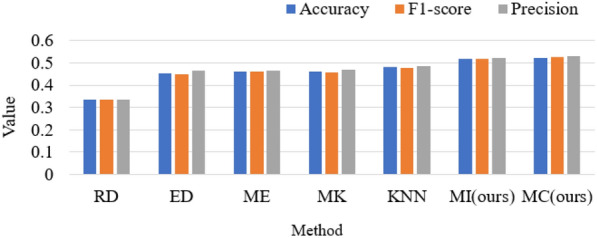
The performance of the proposed model with different kinds of adjacency matrix. RD represents the random, ED represents the Euclidean distance, ME represents Mut_Euclidean, MK denotes the Mut_KNN, MI denotes Mutual Information and MC denotes Mutual_Cos

The results of classification with different kinds of adjacency matrix are shown in Fig. 6.

It can be seen that the MI_cos adjacency matrix has better performance than the MI adjacency matrix, Mul_KNN and Mul_ED are better than KNN and ED which means that mutual information could provide valuable information for ST-GCN. Furthermore, the adjacency matrix surely could effect the performance of classification.

## Conclusion

In this paper, we improve the original model for motor imagery classification task based on our previous work [30]. Instead of using the stable adjacency matrix, we calculate the cosine similarity of the columns of the embedding to generate the dynamic adjacency matrix. The main advantage of the new model is that it could adjust the input matrix during the training process to utilize the features fully. The experiment results demonstrate that the new model outperforms the state-of-the-art methods as well as our previous model. Furthermore, the adjacency matrix has much more impact on the performance of the GCNs, and more suitable adjacency matrix can still be explored.

The current understanding on brain mechanisms is still limited, more influencing factors will be taken into account to further improve the forecasting accuracy. Moreover, motor imagery EEG data present individual differences, such as FBCSP has different performances when experimenting with EEG data that from different subjects, and it can achieve good results when using the same subject’s data for training and testing, but it does not perform well in mixed data of multiple subjects. Individual differences also affected the development of solutions for the classification task of motor imagery. How to eliminate individual differences and extract valuable features is still key for wider application of EEG-based tasks. Some current transfer learning methods may be deployed to eliminate individual differences and further expand the scope of EEG applications.

## Data Availability

The data and code are available upon direct request to the authors.
